# Editorial: Digital technology for tobacco control: Novel data collection, study designs, and interventions

**DOI:** 10.3389/fdgth.2023.1341759

**Published:** 2023-12-01

**Authors:** Lindsey N. Potter, Inbal Nahum-Shani, David W. Wetter

**Affiliations:** ^1^ Center for Health Outcomes and Population Equity (HOPE), Huntsman Cancer Institute, University of Utah, Salt Lake City, UT, United States; ^2^ Department of Population Health Sciences, University of Utah, Salt Lake City, UT, United States; ^3^ Institute for Social Research, University of Michigan, Ann Arbor, MI, United States

**Keywords:** tobacco cessation, just in time adaptive interventions, ecological momentary assessment, intensive longitudinal data, health inequities, digital intervention

**Editorial on the Research Topic** Digital technology for tobacco control: Novel data collection, study designs, and interventions

Tobacco is the leading cause of preventable death and disease and is responsible for nearly one in five deaths in the United States (1–4). Importantly, many tobacco users have a desire to quit, with nearly half of smokers reporting quitting for at least one day in the last 12 months (5, 6). However, traditional tobacco cessation interventions such as self-help materials (7), nicotine replacement therapy (8), and physician advice (9), although efficacious in helping individuals achieve abstinence, can be resource intensive and costly, which could be a barrier for making a population-level impact. Advances in digital technologies have created unprecedented opportunities to leverage novel data collection and intervention designs to improve tobacco prevention and treatment. For example, the use of ecological momentary assessment (EMA) has revealed dynamic predictors of smoking lapse (10–19). Near-continuous GPS data collected from smartphones and physiological data collected from wearable sensors have been used to reveal contextual and physiological precipitants of lapse with more granularity than ever before (e.g., proximity to cues to smoke and autonomic indicators of self-regulatory capacity, which is important for tobacco cessation (20, 21). Importantly, the driving motivation behind the use of these technologies is that derived data can be leveraged to enhance treatment accessibility and scalability, and to deliver adaptive interventions (e.g., Just-in-Time Adaptive Interventions, or JITAIs) (22, 23). To that end, this Research Topic contains 8 articles highlighting (a) data collection approaches that leverage digital technology to gain a better understanding of tobacco use and mechanisms of change; (b) innovative intervention approaches that leverage digital technology to enhance accessibility, scalability, and the individualization of tobacco use prevention and treatment; and (c) research employing novel experimental designs and/or data analytic methods to inform tobacco use prevention and treatment.

Several conceptual pieces offer pragmatic guidance for developing digital interventions. Battalio and colleagues review the social determinants of health (SDOH) that may contribute to tobacco-related health inequities. They present a conceptual model to address SDOH with a lens towards developing mHealth tobacco cessation interventions that are optimized to serve populations most in need (Battalio et al.). Nahum-Shani and colleagues introduce a framework with 5 guiding questions that can be used to select the most appropriate experimental approach (Nahum-Shani et al.). They call for more flexible experimental designs that can efficiently address questions about the integration and adaptation of intervention components at multiple timescales (24, 25). Cui and colleagues highlight the challenges in developing smoking cessation applications for mobile phones, which include sophisticated programming requirements and significant investment of time and money. They provide guidelines for conducting mobile smoking research using Qualtrics and discuss the flexibility, affordability and potential of this approach in facilitating more scalable mobile tobacco cessation interventions (Cui et al.).

Despite the tremendous opportunities that mHealth studies offer for understanding dynamic mechanisms of change and informing interventions, they are especially susceptible to missing data due to challenges relating to participant engagement. Two papers discuss these challenges. Sobolev and colleagues leverage data from two EMA studies of smoking cessation to explore the dynamics of engagement with mobile health data collection in real-world settings. They investigate how engagement with data collection (EMA prompts delivered and EMA prompt response) unfolds over time, and based on the results emphasize the importance of integrating multiple indicators to measure engagement (Sobolev et al.). Ji and colleagues utilize data from an EMA study of smokers attempting to quit, as well as a simulated data set, to demonstrate how improper accommodation of multilevel intensive longitudinal data structures in multiple imputation may impact study results. They emphasize the importance of properly handling clustered missingness for conclusions drawn from ILD studies that are used to inform the development of tobacco cessation interventions (Ji et al.).

Several articles examine dynamic factors that influence tobacco cessation success. Coughlin and colleagues review the state of the science of using motivational incentives in smoking cessation interventions. They highlight the benefits of digitally delivered motivational incentives for reducing barriers associated with smoking cessation interventions, such as participant burden, disengagement, and up-front costs. To help mitigate these barriers, they call for the development of digitally delivered motivational incentive interventions that are guided by several principles for constructing JITAIs, which can enhance the feasibility, effectiveness, and scalability of digital motivational incentive interventions for smoking cessation (Coughlin et al.). Scherer and colleagues examine the time-varying nature of self-regulation in real-world settings in two high-risk populations (individuals who smoke and individuals with binge-eating disorder). They demonstrate that self-regulation is not static, but rather may vary based on contextual factors (e.g., location, environmental cues to smoke, and others), and discuss the implications for interventions targeting momentary self-regulation as a means to reduce health risk behaviors (Scherer et al.).

Finally, Benson and colleagues report on a pilot RCT comparing 3 smoking cessation interventions: a JITAI that tailored treatment in real time, the National Cancer Institute QuitGuide application, and a clinic-based tobacco cessation programing that follow clinical practice guidelines. These interventions target negative affect and urge, factors that influence tobacco cessation in daily life. Based on findings that the within-person association between negative affect and urge was stronger in the post-quit than pre-quit period, and that associations differed by intervention type, the authors discuss the potential importance of personalizing interventions for decoupling momentary associations between negative affect and urge during a quit attempt (Benson et al.).

Included in this Research Topic are original reports highlighting novel frameworks, study designs, and data collection procedures, as well as intervention and methodological approaches that leverage advances in digital technologies to prevent and treat tobacco use. We believe this Research Topic demonstrates that digital technologies offer a tremendous opportunity to leverage information about an individual's progress in treatment, internal state, and context to recommend whether and how to intervene (22), which, in turn, can improve accessibility and scalability of evidence-based interventions, and reduce tobacco-related inequities at the population-level (26).
